# Insecticide Resistance in Eggs and First Instars of the Bed Bug, *Cimex lectularius* (Hemiptera: Cimicidae)

**DOI:** 10.3390/insects6010122

**Published:** 2015-01-15

**Authors:** Brittany E. Campbell, Dini M. Miller

**Affiliations:** 1 Department of Entomology and Nematology, University of Florida, 1881 Natural Area Drive, Gainesville, FL 32611, USA; 2 Dodson Urban Pest Management Laboratory, Department of Entomology, Virginia Tech, 170 Drillfield Drive, Blacksburg, VA 24061, USA; E-Mail: dinim@vt.edu

**Keywords:** bed bug, *Cimex lectularius*, egg, first instar, resistance

## Abstract

Two strains of the common bed bug, *Cimex lectularius* L., eggs and first instars collected from pyrethroid-resistant adults were evaluated for insecticide resistance and compared to a susceptible strain. Dose-response bioassays were conducted using two insecticide formulations (Temprid: imidacloprid/β-cyfluthrin, and Transport: acetamiprid/bifenthrin). The lethal concentration (LC_50_) for the two resistant egg strains exposed to imidacloprid/β-cyfluthrin ranged from 3 to 5-fold higher than susceptible strain eggs. Resistant strain eggs dipped into formulations of acetamiprid/bifenthrin had LC_50_ values which were significantly greater (39 to 1,080-fold) than susceptible strain eggs. Similar to eggs, resistant strain first instars exposed to residual applications of imidacloprid/β-cyfluthrin had LC_50_ values ranging from 121 to 493-fold greater than susceptible strain first instars. When resistant strain first instars were treated with acetamiprid/bifenthrin, they had LC_50_ values that were 99 to >1,900-fold greater than susceptible strain first instars. To determine differences between egg and first instar resistance, stage resistance ratios (SRR) were compared between the two stages. There was little difference between the egg and first instar stages, indicated by small SRR values ranging from 1.1 to 10.0. This study suggests that insecticide resistance is expressed early during bed bug development.

## 1. Introduction

Although studies have documented that bed bugs (*Cimex lectularius* L. and *Cimex hemipterus* F.) can carry multiple pathogenic organisms on their bodies and in their excrement, they are not known to be successful at disease transmission and the risk of disease transmission is negligible [1,2,3]. Subsequently, bed bugs do not have the public health status associated with other blood sucking arthropods, including mosquitoes, ticks and fleas. However, bed bug bites can result in allergic cutaneous reactions in humans with varied symptoms [4,5,6,7,8,9], and large bed bug populations require more frequent blood meals that can result in anemia in the host [10]. Aside from physiological complications brought upon by bed bug infestations, bed bugs also can cause psychological distress including depression, sleeplessness and anxiety [11,12]. Furthermore, bed bugs can be economically devastating because of the high costs associated with their control.

There are a number of factors that make bed bug infestations difficult to control. For example, bed bugs are a cryptic species and hide in household belongings (electronics, books, toys, *etc.*) that cannot be treated with conventional insecticides. Another factor making bed bugs difficult to control is their high resistance to many insecticides currently labeled for bed bug control. Bed bug insecticide resistance is a result of *kdr* mutations, enhanced enzyme detoxification activity and cuticular penetration resistance. The cost of bed bug treatments further complicate bed bug control because many people cannot afford the labor intensive treatments required to eliminate bed bug infestations. Lastly, bed bug eggs contribute to the difficulties and costs associated with bed bug treatments because of their small size and the lack of effective insecticides against them.

Most conventional insecticides labeled for bed bug control are ineffective against bed bug eggs [13]. Conventional bed bug protocols require at least three treatments applied at two week intervals to allow bed bug eggs to hatch. Consequently, pest control professionals are treating newly hatched nymphs rather than the eggs. Although bed bug eggs are difficult to control, there are few studies available that have evaluated insecticide efficacy for controlling bed bug eggs [14,15]. This is the first study to evaluate bed bug egg and first instar resistance.

Insecticide resistance in different species of insect eggs has been demonstrated where resistance was also quantified in the adult or larval stages of the same species [16,17]. Head louse eggs had higher lethal concentration ratio (LCR) values when treated with permethrin compared to head louse adults [16]. Egg mortality was much higher for a susceptible strain of *Plutella xylostella* compared to a resistant strain after treatment with the same concentrations of deltamethrin [17]. This study also evaluated mortality of the emerging larvae of *P. xylostella* treated eggs and found that mortality was lower in the resistant strain larvae compared to the susceptible strain following deltamethrin treatment [17]. Insecticide resistance between eggs and first instars has been shown to be differentially expressed in *Triatoma infestans* [18], suggesting that first instar resistance was not indicative of egg resistance.

The purpose of this study was to determine insecticide resistance in bed bug eggs and first instars. We conducted dose-response bioassays with two combination products commonly used for bed bug control, Temprid SC (imidacloprid [0.10%]/β-cyfluthrin [0.05%], Bayer CropScience, Research Triangle Park, NC, USA) and Transport GHP (acetamiprid [0.05%]/bifenthrin [0.06%]; FMC Corp., Philadelphia, PA, USA) to determine LC_50_ values and subsequent resistance ratios of bed bug eggs from three strains. We also assessed bed bug egg mortality with a pyrethroid insecticide, Suspend SC (deltamethrin [0.06%], Bayer CropScience, Research Triangle Park, NC, USA) which has been used for several years for bed bug control. Consequently, several studies have documented deltamethrin resistance in adult bed bugs [19,20,21,22,23] but not in other bed bug life stages.

## 2. Experimental Section

### 2.1. Experimental Insects

Three bed bug, *Cimex lectularius*, strains were used for this study, a pyrethroid-susceptible strain (Harlan), and two pyrethroid-resistant field strains (Richmond and Epic Center). The Harlan susceptible strain was acquired from Dr. Harold Harlan (National Pest Management Association, Fairfax, VA, USA) in February 2005. The Richmond resistant strain was collected from an elderly group home located in Richmond, VA in 2008 and has been found to be highly resistant to pyrethroid insecticides [21]. The Epic Center resistant strain was collected in 2008 from an apartment complex in Cincinnati, Ohio. Epic Center adult bed bugs confined on dried deltamethrin (0.06%) residues had a resistance ratio of 392 when compared to the Harlan susceptible strain.

All bed bug strains were fed weekly with defibronated rabbit blood (Hemostat, Dixon, CA, USA) on an artificial feeding system. The bed bug strains were maintained in plastic rearing jars enclosed with mesh at one end to allow for feeding. Rearing jars contained pieces of cardboard to provide harborage and a substrate for the bed bugs to walk up and feed through the mesh. The plastic rearing jars containing all bed bug strains were stored in an environmental chamber at 27 °C, 60% RH, and a 12:12 L:D photoperiod.

Prior to the bioassay, recently fed and mated female bed bugs (30 groups of 10) were collected from all three strains and placed into plastic Petri dishes (Fisher Scientific Inc., Waltham, MA, USA; 6 cm × 5 cm) each containing a piece of filter paper (Whatman # 1; 4.2 cm diameter ) for oviposition. The females were provided a new piece of filter paper daily. 

### 2.2. Epic Center Adult Resistance Assessment

Hardboard panels (7 cm^2^) were sprayed to the point of runoff with deltamethrin (0.06%) at the label rate and then allowed to dry completely (~3 h). Control panels were treated to the point of runoff with tap water. Five replicates of 10 adult males were fed 7 days prior to testing and removed from rearing jars and placed inside plastic Petri dishes (35 × 10 mm) 1 day prior to testing. Once the panels were dry, the Petri dishes containing bed bugs were inverted onto the treated surface of the panel and mortality was recorded at regular timed intervals. Control mortality was corrected for using Abbott’s formula:

$$\left(\frac{\%\;test\;mortality\;-\;\%\;control\;mortality}{100\;-\text{ \% }control\;mortality}\;\times \;100\right)$$.

### 2.3. Egg Resistance Assessment

Bed bug eggs (4–5 days old) were removed from filter papers using soft-tip forceps. Egg removal caused no visible damage to the eggs and did not result in increased mortality compared to a control group of eggs that were not removed from filter papers. The selected age range (4–5 days) was chosen for the bioassay to allow maximum embryonic development while simultaneously avoiding hatch during the test.

Three insecticides were chosen for this resistance evaluation: (1) Temprid SC; (2) Transport GHP; and (3) Suspend SC. All of these products were chosen because they are routinely used for bed bug treatments in the United States [24]. All eggs were exposed to five concentrations of each insecticide formulated with water ranging from 0.21–21 µL/mL for imidacloprid/β-cyfluthrin and 0.004–33.8 ng/mL for acetamiprid/bifenthrin. Control treatment eggs were dipped into water only and control mortality was corrected for using Abbott’s formula.

Bed bug eggs (5 replications of 10) were dipped into each insecticide concentration using a centrifuge tube (Fisher Scientific Inc., 50 mL) that had been cut in half. A large hole was cut into the lid and covered with mesh. The eggs were placed onto the mesh closure of the centrifuge tube and immersed into each insecticide formulation for 5 s. The mesh, containing eggs, was then dried with a KimWipe (Kimberly-Clark Professional, Roswell, GA, USA; 11 cm × 21 cm) to remove excess insecticide. Using a paint brush, eggs were immediately removed from the mesh into a plastic Petri dish (BD Falcon, Durham, NC, USA; 50 × 9 mm) containing a clean piece of filter paper. Egg hatch failure was determined by no first instar emergence and recorded 14 days post treatment. 

### 2.4. First Instar Resistance Assessment

Harlan, Richmond, and Epic Center strain bed bug eggs were allowed to hatch within plastic Petri dishes (BD Falcon, 50 × 9 mm). Following hatch, unfed first instar bed bugs (7–10 days old) were collected using a paint brush. Formulations of both imidacloprid/β-cyfluthrin (Temprid SC) and acetamiprid/bifenthrin (Transport GHP) were used for this study. All three strains of first instars were placed onto dried, treated surfaces saturated with one of the five concentrations of each insecticide, ranging from 0.007–21 µL/mL imidacloprid/β-cyfluthrin and 0.004–33.8 ng/mL acetamiprid/bifenthrin. An aliquot (150 µL) of each insecticide concentration was applied to a filter paper disc (Whatman # 1; 4.2 cm diameter) and allowed to dry completely. The 150 µL aliquot of insecticide fully covered the filter paper but did not saturate the paper to the point of runoff. The treated filter papers were then placed on top of a hardboard panel (7 cm^2^). Control treatments received only water and control mortality was corrected for using Abbott’s formula.

First instars (5 replications of 5 insects) were released on top of the treated surface and contained by inverting the bottom of a plastic Petri dish (BD Falcon, 50 × 9 mm) on top of the treated filter paper. The Petri dish was smaller in diameter than the filter paper, therefore all of the first instars were continuously exposed to the treated surface. To ensure that first instars could not escape, small metal weights were placed on top of the plastic Petri dishes. Mortality was recorded after 24 h and was defined by individuals that did not move after prodding with a paint brush after 24 h.

### 2.5. Statistical Analysis

The LC_50_ values (concentration that kills 50% of individuals) were calculated for eggs and first instars from each strain exposed to each insecticide using Probit analysis [25]. Deltamethrin LC_50_ values were not calculated for bed bug eggs because there was little bed bug egg mortality at the highest tested concentrations. Significant differences between the three strains exposed to deltamethrin were determined by ANOVA and *p*-values ≤ 0.05 were used to indicate significance (JMP^®^ Pro 10.0 software; SAS institute, Cary, NC, USA). Due to low bed bug egg mortality with deltamethrin (0.06%), we did not test first instars using deltamethrin.

Significant differences between LC_50_ values of eggs and first instars from each strain exposed to each insecticide were determined by the failure of the confidence intervals (CI) to overlap. LC_50_ values were calculated using PoloPlus [25]. Resistance ratios were calculated by dividing the LC_50_ value of the resistant strain by the LC_50_ for the susceptible strain for both eggs and first instars. To further evaluate differences between egg and first instar resistance, we calculated stage resistance ratios. These stage resistance ratios were determined by dividing the largest LC_50_ value of either stage (egg or first instar) by the smallest LC_50_ value of either stage.

## 3. Results

### 3.1. Epic Center Strain Resistance Assessment

Epic Center adult bed bugs were found to be less susceptible to deltamethrin (0.06%) compared to the Harlan susceptible strain, indicated by the high resistance ratio (Harlan: LT_50_ = 1.06 h; 95% CI = 0.83–1.38, Epic Center: LT_50_ = 415.98 h; 95% CI = 352.72–517.43; RR = 392). Previous studies have established resistance in Richmond adult bed bugs [19,21]. Therefore, this experiment was to establish that the Epic Center strain was resistant for subsequent bed bug egg and first instar evaluations.

### 3.2. Egg Resistance Assessment

**Figure 1:**
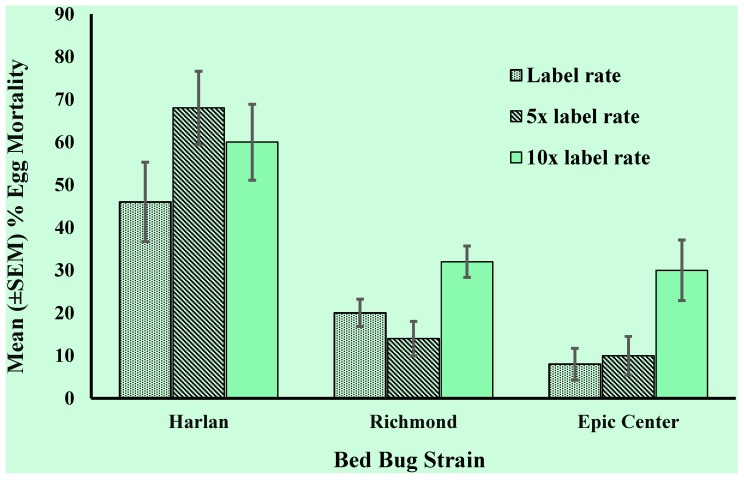
Mean (±SEM) bed bug egg percent mortality of a pyrethroid-susceptible strain (Harlan) and two pyrethroid-resistant strains (Richmond and Epic Center) after treatment with deltamethrin (0.06%). Five replications of 10 bed bug eggs were dipped into three concentrations of deltamethrin for a total of *n* = 50 at each tested concentration.

Lethal concentration values (LC_50_) could not be calculated for bed bug eggs treated with deltamethrin because we could not formulate a concentration high enough to cause ≥80% mortality that would stay in suspension. Richmond and Epic Center resistant strain eggs had significantly lower percent mortality (*p* ≤ 0.0001) when exposed to deltamethrin compared to Harlan susceptible strain eggs at all tested concentrations (Figure 1). As expected, Harlan strain susceptible eggs died at lower concentrations than the other two bed bug egg strains tested when exposed to imidacloprid/β-cyfluthrin (LC_50_ = 0.41 µL/mL) and acetamiprid/bifenthrin (LC_50_ = 0.02 ng/mL) (Table 1). Richmond and Epic Center eggs were not highly resistant to imidacloprid/β-cyfluthrin (Richmond RR = 3.0; Epic Center RR = 5.1), although the LC_50_ values of both strains were significantly greater than that of the Harlan strain (Table 1). However, Richmond and Epic Center eggs were much more resistant to acetamiprid/bifenthrin, indicated by relatively high resistance ratio values (Richmond RR = 39; Epic Center RR = 1,080). The Epic Center strain LC_50_ value was significantly greater than Richmond when exposed to acetamiprid/bifenthrin, as indicated by non-overlapping confidence intervals.

### 3.3. First Instar Resistance Assessment

The LC_50_ values calculated for Richmond and Epic Center first instars were significantly greater than that of the Harlan strain first instars when exposed to imidacloprid/β-cyfluthrin, but were not significantly different from each other (Table 2). However, the calculated resistance ratios for Epic Center eggs was 4-fold greater than that of the Richmond strain eggs when exposed to imidacloprid/β-cyfluthrin (Table 2). The LC_50_ value for Harlan first instars (0.007 ng/mL) was significantly lower than the other two strains when exposed to acetamiprid/bifenthrin. The LC_50_ values were significantly different between all three strains when treated with acetamiprid bifenthrin; Harlan < Richmond < Epic Center (Table 2). The resistance ratio values calculated for Epic Center strain first instars exposed to acetamiprid/bifenthrin was 20-fold greater than that of Richmond strain eggs.

**Table 1:** Comparison of bed bug egg LC_50_ values when exposed to 5 different concentrations of imidacloprid/β-cyfluthrin and acetamiprid/bifenthrin for a pyrethroid-susceptible strain (Harlan) and two pyrethroid-resistant strains (Richmond and Epic Center).

Strain	*n*	LC_50_ (95% CI)	Slope ± SE	*X*^2^ (*df*)	RR
Imidacloprid/β-cyfluthrin					
Harlan	250	0.41 μL/mL (0.28–0.55) ^b^	1.86 ± 0.24	33.42 (23)	
Richmond	320	1.23 μL/mL (0.59–2.10) ^a^	1.13 ± 0.14	82.57 (30)	3.0
Epic Center	400	2.10 μL/mL (1.05–4.59) ^a^	0.95 ± 0.10	149.91 (38)	5.1
Acetamiprid/bifenthrin					
Harlan	250	0.02 ng/mL (0.02–0.03) ^c^	2.33 ± 0.25	26.90 (23)	
Richmond	310	0.78 ng/mL (0.37–1.44) ^b^	0.58 ± 0.10	29.17 (29)	39
Epic Center	240	21.6 ng/mL (6.4–51.3) ^a^	0.48 ± 0.09	25.71 (22)	1080

LC_50_ values followed by different letters are significantly different determined by the failure of the confidence intervals to overlap [25].

**Table 2:** Comparison of bed bug first instar LC_50_ values when exposed to 5 different concentrations of imidacloprid/β-cyfluthrin and acetamiprid/bifenthrin for a pyrethroid-susceptible strain (Harlan) and two pyrethroid-resistant strains (Richmond and Epic Center).

Strain	*n*	LC_50_ (95% CI)	Slope ± SE	*X*^2 ^(*df*)	RR
Imidacloprid/β-cyfluthrin					
Harlan	150	0.04 µL/mL (0.03–0.06) ^b^	2.16 ± 0.34	38.17 (28)	
Richmond	195	4.81 µL/mL (1.94–10.26) ^a^	0.66 ± 0.12	45.87 (37)	121
Epic Center	190	19.72 µL/mL (8.18–184.48) ^a^	0.75 ± 0.17	45.39 (36)	493
Acetamiprid/bifenthrin					
Harlan	155	0.007 ng/mL (0.005–0.008) ^c^	3.29 ± 0.48	33.95 (29)	
Richmond	125	0.69 ng/mL (0.21–1.43) ^b^	0.94 ± 0.19	29.91 (23)	99
Epic Center	115	13.6 ng/mL (3.9–1215.8) ^a^	0.50 ± 0.13	23.31 (21)	1943

LC_50_ values followed by different letters are significantly different determined by the failure of the confidence intervals to overlap (PoloPlus 2004).

### 3.4. Stage Resistance Comparisons

Harlan eggs were slightly less susceptible than Harlan first instars (Stage resistance ratio [SRR] = 3.3) when treated with acetamiprid/bifenthrin (Table 3). Harlan eggs were even less susceptible than first instars when treated with imidacloprid/β-cyfluthrin (SRR = 10.0). Richmond first instars were less susceptible than Richmond eggs to imidacloprid/β-cyfluthrin (SRR = 3.9). Epic Center first instars were also less susceptible to imidacloprid/β-cyfluthrin than Epic Center eggs (SRR = 9.4). There was relatively no difference between Richmond and Epic Center eggs and first instars exposed to acetamiprid/bifenthrin, indicated by a stage resistance ratio close to 1 (Table 3).

**Table 3:** Comparison of stage resistance ratios (RR) between eggs and first instars within strain (Harlan pyrethroid susceptible, Richmond pyrethroid resistant and Epic Center pyrethroid resistant). Eggs and first instars were treated with either imidacloprid/β-cyfluthrin or acetamiprid/bifenthrin.

Strain	Stage with > LC_50_	Stage RR
Imidacloprid/β-cyfluthrin		
Harlan	egg	9.98
Richmond	1st instar	3.91
Epic Center	1st instar	9.4
Acetamiprid/bifenthrin		
Harlan	egg	3.28
Richmond	egg	1.13
Epic Center	egg	1.51

Stage resistance ratios were determined by dividing the largest LC_50_ value of either stage (egg or 1st instar) by the smallest LC_50_ value of either stage. The stage (egg or first instar) with the greater LC_50_ value is indicated in the >LC_50_ value column. LC_50_ value information for both stages is presented in Table 1 and Table 2.

## 4. Discussion

Most research evaluating pyrethroid insecticide efficacy in bed bugs has focused on third instars and subsequent life stages [19,20,21,22,23]. Goddard [14] evaluated the efficacy of several insecticide products on bed bug eggs but did not evaluate egg resistance. Bed bug egg mortality could be achieved with some pressurized aerosol insecticides but the same active ingredients formulated in water lacked efficacy against bed bug eggs [14]. Surprisingly, when we tested deltamethrin at 10× the label rate against susceptible strain bed bug eggs, we did not achieve 100% mortality. The lack of efficacy of deltamethrin for Harlan susceptible eggs may be a result of the waxy components of the eggshell preventing water formulated products from permeating the eggshell. Insect eggs are known to differ in their susceptibility to insecticides compared to other stages in the insect life cycle [26]. These differences in susceptibility are attributed to the inherent properties of an insect egg, including adaptation of the eggshell for oxygen intake, the development of the nervous system and other physiological systems, and the multiple layers that comprise the eggshell. Insecticide efficacy is highly influenced by the permeability of the eggshell layers that surround the embryo [26]. Although the embryo inside of the Harlan egg may be susceptible, the insecticide still had multiple barriers to penetrate associated with the eggshell before reaching the target site, thus resulting in lower mortality in the susceptible strain than expected.

Richmond adult bed bugs are known to be resistant to pyrethroid insecticides [19,21]. We also documented resistance in adult Epic Center bed bugs. Comparisons of bed bug egg resistance ratios to adult resistance ratios would be ideal, but we could not achieve enough egg mortality at even the highest tested concentration of deltamethrin to calculate LC_50_ values. However, eggs from both Richmond and Epic Center strains had low mortality compared to eggs from the Harlan susceptible strain when exposed to the same concentrations of deltamethrin (*p* < 0.0001), indicating that the Epic Center and Richmond eggs may be deltamethrin resistant.

New insecticide products have combined a pyrethroid insecticide with a neonicotinoid in attempts to circumvent the widespread resistance to pyrethroid products. Pest control operators in the United States surveyed in 2011 routinely used the combination pyrethroid/neonicotinoid products, Temprid and Transport, for bed bug treatments [24]. Potter *et al.* [27] compared the efficacy of Temprid and Transport to Suspend (deltamethrin; 0.06%) via direct spray and residual applications, and found that both combination products were more effective against adult bed bugs than deltamethrin.

Overall, bed bug eggs and first instars from Richmond and Epic Center strains were somewhat resistant to the imidacloprid/β-cyfluthrin combination product but were more resistant to the acetamiprid/bifenthrin combination product, with the exception of Richmond first instars. We did not determine if the observed resistance is to the neonicotinoid or the pyrethroid component because we tested combination products. In 2008, when these bed bug populations were collected in the field, pest control companies were primarily using only pyrethroid products for chemical control. Temprid SC was not even labeled for bed bug control until 2010 (bed bug label amendment 14 January 2010; EPA registration No. 432–1483). Although FMC registered Transport GHP in 2008, it is unlikely that Richmond and Epic Center bed bug populations had been exposed to Transport GHP.

Resistance has been previously documented to both combination products we tested, imidacloprid/β-cyfluthrin and acetamiprid/bifenthrin, in adult bed bug populations [28]. Furthermore, insecticide resistance has been documented to be highly variable between different bed bug populations [20,28]. The bed bug populations we tested varied in their levels of susceptibility. The Richmond and Epic Center strains we tested were collected from different geographic locations within the United States. Therefore, differences between resistance in Richmond and Epic Center eggs could be a result of previous insecticide exposure and selection pressure.

Interestingly, comparisons of stage resistance ratios indicated that there was little difference in resistance between eggs and first instars. The eggshell may provide some protection from insecticides but is not the likely determinant factor in egg resistance. The observed resistance in the first instars further establishes that the eggshell is not the only determinant factor in resistance but the embryo (which will be the emerging first instar) already has developed resistance at an early age of bed bug development.

## 5. Conclusions

Overall, this research suggests that resistance is expressed early in the bed bug life cycle and that first instars are similarly resistant to insecticides as bed bug eggs. The eggshell is probably providing a barrier for insecticide penetration, but the embryo inside of the egg may also have similar resistance mechanisms as documented in adult bed bugs. The insecticide must first penetrate the eggshell, possibly through the respiratory structures (aeropyles), or through openings that allow fertilization of the egg (micropyles), or it must penetrate the multiple chorionic layers of the eggshell. Once the insecticide penetrates the eggshell, it must still overcome resistance mechanisms of the embryo before reaching the target site. Early development of insecticide resistance in the bed bug life cycle should be considered when developing management strategies for bed bug control.
